# Bacteriophages as Solid Tumor Theragnostic Agents

**DOI:** 10.3390/ijms23010402

**Published:** 2021-12-30

**Authors:** Srivani Veeranarayanan, Aa Haeruman Azam, Kotaro Kiga, Shinya Watanabe, Longzhu Cui

**Affiliations:** Division of Bacteriology, Department of Infection and Immunity, School of Medicine, Jichi Medical University, Shimotsuke-shi 3290498, Japan; srivani@jichi.ac.jp (S.V.); azamkla12@jichi.ac.jp (A.H.A.); k-kiga@jichi.ac.jp (K.K.); swatanabe@jichi.ac.jp (S.W.)

**Keywords:** bacteriophages, theragnostics, tumor

## Abstract

Cancer, especially the solid tumor sub-set, poses considerable challenges to modern medicine owing to the unique physiological characteristics and substantial variations in each tumor’s microenvironmental niche fingerprints. Though there are many treatment methods available to treat solid tumors, still a considerable loss of life happens, due to the limitation of treatment options and the outcomes of ineffective treatments. Cancer cells evolve with chemo- or radiation-treatment strategies and later show adaptive behavior, leading to failed treatment. These challenges demand tailored and individually apt personalized treatment methods. Bacteriophages (or phages) and phage-based theragnostic vectors are gaining attention in the field of modern cancer medicine, beyond their bactericidal ability. With the invention of the latest techniques to fine-tune phages, such as in the field of genetic engineering, synthetic assembly methods, phage display, and chemical modifications, noteworthy progress in phage vector research for safe cancer application has been realized, including use in pre-clinical studies. Herein, we discuss the distinct fingerprints of solid tumor physiology and the potential for bacteriophage vectors to exploit specific tumor features for improvised tumor theragnostic applications.

## 1. Introduction

Cancer remains the third leading cause of death after cardiovascular and infectious diseases and it comes as no surprise that one out of four deaths in developed countries is due to cancer. Decades of research has significantly led to a deep understanding of the biology of cancer, its causes, and prospective therapies. Despite many positive results, cancer remains lethal, even in this modern era, with 17 million new cases and 9.5 million deaths in 2018. The global burden is estimated to rise to an alarming 27.5 million new cancer cases by 2040, highlighting the urgency of discovering newer and effectual treatment strategies [1,2,3]. The current treatment options (surgery, radiation therapy and chemotherapy) are limited due to: (1) inability to cross biological barriers, (2) non-specific side effects, (3) minimal effects on metastatic tumors, (4) multi-drug resistance, and (5) lack of efficient diagnostic/treatment examining procedures [4]. Assessment of the disease, such as type, stage, and development, decides the modality of therapy, though chemotherapy accompanied by surgery/radiation is the most utilized and preferred method for treatment of local or metastatic tumors alike, which is fine-tuned by clinicians, depending upon the patient’s condition and medical history.

Cancer is not a simple disease, rather a complex entity which affects any organ or tissue type in the body [5]. It has a genetic predisposition—accretion of mutations in cell cycle check point genes. The mutation in cell cycle check points leads to self-reliant unrestrained immortal cell growth and multiplication, invading adjacent tissues/organs, and metastasis to distant sites. Vigorous variations in the cell’s genome, importantly the gain-of-function mutations of oncogenes and loss-of-function mutations in tumor suppressor genes have been identified in many naturally occurring human and animal tumors and in experimental lab animals [6]. Phenotypically, cancer cells show an irregular cell shape with multi-nucleation that carries coarse and aggregated chromatin distribution. A small cytoplasmic volume with multiple enlarged nucleoli is also identified in many solid tumor cells. Another striking feature of tumor cells is their ability to stay immature and undifferentiated, while the counterpart normal cells mature into specialized cells. A further notable feature is the presence of too many disorganized angiogenic blood vessels [4,5,6]. Most of the present cancer treatment strategies encounter drawbacks in one or more aspects. The main drawbacks of conventional chemotherapies are poor drug distribution, non-specific toxicity, reduced circulation time, tumor relapse, etc. [7,8,9]. Though many promising drugs with anti-cancer effects are identified, a proper carrier system for target-specific therapy is still lacking. An evolving class, nanotechnology-based therapy, is gaining momentum due to its ability to overcome the aforementioned conventional challenges. Nanoscale agents, like nanomaterials, serve as efficient vectors in transporting cytotoxic agents to target sites with the help of targeting strategies/via the enhanced permeability retention (EPR) effect [10,11,12,13,14]. Another class of prospective agents are microbe-based theragnostic agents, especially oncolytic viruses [15,16,17,18,19]. However, with issues pertaining to the pathogenic nature of human-infecting viruses, an alternative that utilizes bacteriophages as theragnostic agents is being considered [20,21]. Bacteriophage theragnostic carriers form an emerging interdisciplinary field that utilizes non-infectious viral vectors as carriers for various therapeutic moieties. In this review, we will discuss briefly the distinct characteristics of solid tumor physiology, and how the tumor cells differ from normal cells. This section is necessary in designing/developing suitable bacteriophage vectors that can exploit the unique characteristics of tumors to target and impart efficacy without disturbing the normal cells, thus minimizing unwanted side effects. In the next section, we discuss how bacteriophages are utilized to ferry therapeutics/diagnostic tools to solid tumors. In the following section, we discuss how variations in tumor physiology are being exploited for developing better phage theragnostic agents, drawing on accessible literature.

## 2. Solid Tumor Physiology

**Figure 1:**
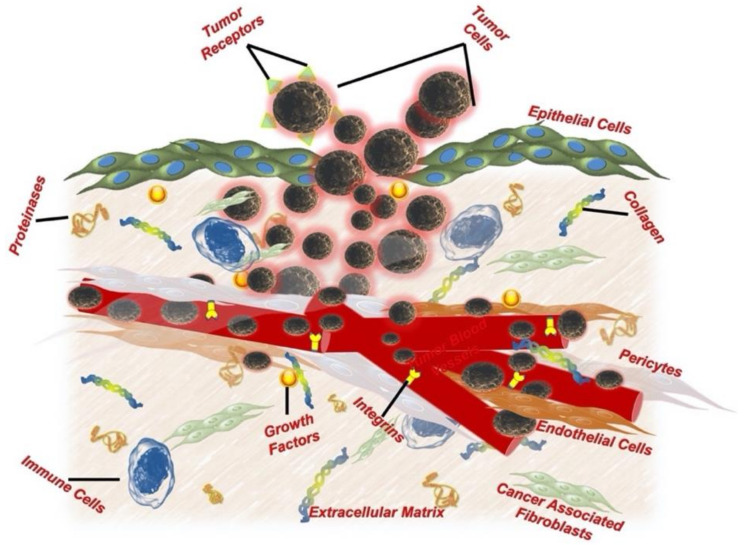
Solid tumor’s microenvironmental architecture.

Cancer, vitally different from adjacent tissue, grows uncontrollably in a specific conditioned environment known as the cancer microenvironment. This niche provides the essentials for clonal expansion and barricades access to the tumor core from external biological factors. This specialized environment is a complex organization of various physical, chemical, and biological players that work together to nurture the growing tumor. The tumor microenvironment is well known for its cellular heterogeneity, comprising stromal cells, fibroblasts (cancer-associated fibroblasts), angiogenic endothelial cells, and tumor-associated immune cells. The other major non-cellular components of this niche include the extracellular matrix (ECM), matrix metalloproteinase (MMP), tumor growth factors and receptors. In addition to biological entities (cellular and non-cellular components), physical and chemical factors such as acidosis, hypoxia, and interstitial fluid pressure also differentiate the tumor microenvironment from a normal cell niche [22,23,24,25,26,27,28,29]. These factors collectively interact within themselves and nurture tumor promotion, progression, and metastasis (Figure 1).

## 3. How Cancer Differs from Normal Tissue

Cancer, being a complex disorder that arises due to genetic instability, functions on its own. Nonetheless, cancers possess several striking dissimilarities when compared to normal tissue, that can be utilized as loopholes for targeting the disease. The major features that are specific to cancer are the EPR effect, hypoglycemic acidic niche, dilated and leaky vasculature, and abnormal lymphatics [30]. Due to the dilated, leaky, and tortuous vasculature, tumors exhibit passive accumulation of macromolecules at their site for an extended time due to EPR. The leakage in blood vessels and malfunction thereof leads to failure in rapid macromolecule clearance [31]. Due to a high metabolic rate, the cancer niche is acidic and hypoglycemic. Owing to EPR, the respiratory and metabolic end-products cannot be cleared from the tumor niche, adding to the highly acidic and hypoglycemic condition [32]. Tumors are also known for the expression of tumor-specific antigens/receptors on their cell surface that could be used for active and selective targeting of the tumors, sparing normal cells [33]. In addition, there is a striking difference reported between the normal and tumor cell plasma membrane. The dynamic cell membrane plays a very essential role in regulation of cell survival. Cancer cells re-organize their plasma membrane to help in the process of uncontrolled proliferation, escaping programmed cell death and resisting anti-cancer drug penetration. This phenomenon is attributed to the increased level of total cholesterol and decreased low density lipoprotein in the plasma membrane assembly. These changes in the lipid composition render the cell membrane rigid and less permeable, helping in cancer cell survival and in multi-drug resistance (MDR) [34]. Cancer cells acquire certain special abilities/hallmark features during their progression. These hallmarks are the organizing principle that rationalize the complexity of the disease. The major distinctive features of cancer are accumulating mutations in gate-keeper genes, deregulation of cell metabolism, sustained proliferation, evading growth suppression, evading immune capture, inducing tumor-promoting inflammation, resisting apoptosis, unrestrained replication potential, inducing neoangiogenesis, and establishing invasion and metastasis. These key features of cancer and its microenvironment can be utilized to develop efficient theragnostic approaches that are more specific and selective towards cancer.

## 4. Phages for Cancer Diagnosis and Therapy

Bacteriophages are promising theragnostic options owing to their nanosize, polyvalent surface properties and non-pathogenic nature and are open to desirable chemical or genetic modifications. They are modest uniform biologics made of repetitive units of same coat protein and can be simply prepared in a bacterial host [35]. These properties of bacteriophages make them an attractive biomedical tool for therapeutic as well diagnostic applications. Their macromolecular protein heads serve as an ideal framework for attachment of a wide variety of cargoes such as drugs and fluorescent probes [36]. When suitably surface-functionalized, these phages display enhanced tumor permeability and retention owing to their nanosize effect, which is additionally the reason they can stay clear of the reticulo-endothelial system (RES), spleen, kidney, and hepatobiliary clearance [37,38]. The bacteriophages can be personalized for therapy with drugs/drug cocktails/targeting ligands-antagonists, etc., making them versatile multifunctional entities. Owing to their size, they are also able to cross biological barriers such as the blood brain barrier for treatment of brain tumors and the hypovascular fibrotic barrier for treatment of pancreatic cancer [39]. The surface to volume ratio is high and so these carriers can carry loads of therapeutics to cancer efficiently when compared to macromolecular chemotherapeutics.

## 5. Key Features of Natural Phages

***Abundance:*** Bacteriophages are the most abundant and ubiquitous organisms found in nature and contribute greatly to global genetic diversity [40]. Yet only a handful of them are well-characterized, leaving a broad scope to discover new ones that could be useful for varied biomedical applications. Phages are also prevalent in the human body and are natural predator to microbiomes seen in skin, oral cavity, lungs, intestines, and the urinary tract [41]. They play an important role in maintaining bacterial population dynamics in the human body. When it comes to the human gut microbiome, phages are the most common constituent and are known to utilize leaky gut or Trojan horse mechanisms to access the human body from the gut [42]. Leaky gut is the inflamed intestine due to infection that leads to a dysfunctional intestinal barrier with compromised permeability. Phages are known to access the human body through the punctured vasculature at the inflamed area of leaky gut. The Trojan horse mechanism involves phage-infected bacteria being engulfed by intestinal epithelial cells, thereby giving the phage the ability to cross the intestinal barrier and access the human body. Phages do not have inherent mammalian/human cell tropism, yet they can efficiently transcytose human tissues and are often detected in human blood [43]. It was recently reported that phages can functionally transcytose across cell layers and this study also estimated that approximately 31 billion phages transcytose through the gut into the body every day [44]. Further, many reports suggest the infiltration of phages into major organs like lung, liver, kidney, spleen and even brain, indicating their ability to cross the blood brain barrier [45]. Due to the abundance of phages in nature and the fact that many bacteriophages are unexplored until now, there is a broad prospect available to examine them for their potential use as cancer theragnostic carriers. Bacteriophages, if appropriately exploited and scrutinized in detail for their anti-cancer applications in the way that researchers have explored nanomedicine field, may become one of the significant tumor theragnostic vectors in future.

***Safe andnon-pathogenic nature:*** Bacteriophages are usually considered as safe to humans. They co-exist in the human body in large numbers and with their involuntary presence, effectively tackle and kill numerous infectious bacterial pathogens [41]. Phages are also credited for their antibacterial functions in the human body and with upholding the bacterial population dynamics [42]. Though they are omnipresent in human organs, surprisingly, they produce no significant toxicity to humans. Many pre-clinical and clinical trials have confirmed the safety and tolerability of different phage therapeutics based on different modes of administration [46]. This safety is a valued characteristic when it comes to vectors. If the vectors are themselves toxic, there is a high chance of them being detrimental to cells that come in contact upon administration into the patient. Thereby, bacteriophages can be considered as apt therapeutic vectors, owing to their inert nature to mammalian cells. 

***Size:*** Most phages range in the nanoscale diameter (the smallest known phage has a 40 nm head; average ones range from 70 nm–200 nm, except filamentous phages that are generally few micrometers in length) [47] and are considered as optimal sized vectors/probes for biomedical applications, including cancer theragnostics. Due to their size, though, they are usually processed and cleared by the RES or hepatobiliary system; still, they are perfectly sized to cross the leaky vasculature in tumors but not the normal blood vessel fenestrations, thereby limiting their off-site diffusion. There are many reports that elucidate the ability of the phages to cross the leaky blood vessel and reach the tumor site effectively to impart therapeutic function. This is a customary feature expected for therapeutic cancer vectors, indicating that phages too can be used as passive targeting cancer vectors. However, passing through biological barriers is not easy and a basic understanding of barrier biology can help one develop a phage agent that can pass these barriers more efficiently for a greater therapeutic/diagnostic reach. 

***Phage surface properties:*** The surface charge of any material/macromolecule determines the in vivo pharmacological kinetics [48]. Though not much study has been carried out on surface charge properties of phages, phages with negative charges (−30 mV to −10 mV) at physiological pH [49,50] may have moderately good blood circulation in vivo. In addition, because phages resemble protein nanocarriers, the properties of the protein nanocarriers would suit the phages too [51]. Highly negative charged nanocarriers are usually repelled by negatively charged blood cells and vascular cells whereas high positive/negative charge carriers have been shown to be cleared by the RES system [52]. Use of sterically stabilized carriers with neutral or slight negative or positive charges tend to show extended half-life and negligible clearance by RES, kidney, spleen, or hepatobiliary organs [53]. Phages that exhibit slightly negative charge in physiological pH can thus be employed as vectors for tumor theragnostic use. In addition, steric stabilization like PEGylation of phages could improve the half-life of the phages. 

***Phage surface architecture:*** Phages in general lack diversity in their surface architecture and their surfaces are mostly made up of conserved proteins. In addition, they are simple nucleocapsids, solely made of nucleic acids and proteins [54]. This simpler architecture of the phages, with defined and repetitive structural units [55], may yield analogous immune response among the closely related phages upon in vivo administration. As the immune response tends to not change much for closely related phages, the response is easily predictable and could be made adaptable by similar types of surface modification that could be extended to those closely related phage members. PEGylation of phages is one such approach that is proven to reduce the immune response against the phages when administered in vivo [37].

***Phage stability:*** Phages are reported to be stable in wide pH and temperature conditions [56]. One of the stability analysis studies established that phages are stable at pH 3 to 11 for a 24 h period. In addition, phages also remained stable when freeze-dried, lyophilized, desiccated, and through repeat freeze-thaw cycles [57]. Another study established the thermostability of the phages at 63 °C for up to 6 weeks [58]. A recent study also established the optimal stability of phages in different buffered infusion solutions that are commonly used in medical treatment [59]. 

***Phage clearance:*** The most serious limitation of using phages as a theragnostic carrier is their rapid clearance by the RES, that decreases their blood half-life considerably [60]. This clearance by RES is an expected outcome considering the size of the phages. Most research on phage clearance point to RES as being responsible for clearing the phages from circulation. Post clearance, the phages are seen concentrated in the spleen, liver and other RES organs and are rapidly processed by splenic or liver macrophages for elimination from the system [61]. However, repeated administration of phages in circulation has resulted in certain variants displaying better blood half-life and these are known as long-circulating phages. These phage variants are mutants that mostly carry single mutations in their capsids which contribute to evading the RES clearance effectively while significantly extending the circulation time compared to natural phages [62]. Another striking feature of the phages is that they are seen in the urine samples of humans, that indicate that phages can be processed by renal filtration also [41]. 

***Immune response to phages:*** Phages are known to modulate innate as well as humoral immunity. In case of innate immunity, phages can induce cytokine response post phagocytosis into immune cells. Post phagocytosis into immune cells (dendritic cells, macrophages), the surface pathogen-associated molecular patterns (PAMPs), owing to the naturally antigenic coat proteins of the phage head and the CpG islands in the phage genome, induce innate immune response [63]. Next, phages exert their effect in adaptive immunity by inducing antibody production, activation of T helper cells, and effector polarization [64]. Naturally occurring bacteriophages are known to activate antibody production against themselves; phage neutralizing antibodies were found in the sera of humans upon phage exposure [65]. Spleen, one of the organs of RES, plays an important role in the production of anti-phage antibodies [66]. Related bacteriophages carry similar antigens and thus antibodies produced upon antigen presentation and stimulation can cross-react with related phages [67,68].

## 6. Amenable Phage Features

***Phage display:*** Phage display is the well-established molecular technique that explores the fusion of foreign peptides/proteins on coat proteins of the virion. These displayed identities sit in the assembled virions and are available to external milieu. There have been a variety of applications such as immunotherapy and bio-panning using this technology [69,70,71]. The bio-panning can be extended to tumor targeted therapy as it can be used to isolate peptides that bind to cancer cell-specific receptors/markers [72]. Another approach is to display known tumor-targeting peptides on the phage virion, thereby introducing mammalian cell tropism which could eventually render them adaptable for cancer-targeted applications. This method is a boon to targeted therapy as the natural phages can be genetically engineered to display targeting peptides on their surface by simple yet well-established techniques. Once bound to a cancer cell’s receptors, these agents are endocytosed, leading to entry of theragnostic agents and action thereof. This strategy helps in precision therapy that spares normal cells as well as aiding in endocytosis and release of the therapeutic agent in the cancer cell’s cytosol [73]. This is a cost-effective and less-time consuming method of developing targeted therapeutics when compared to chemical or physical conjugation of targeting moieties onto nanoparticles or on molecular drugs, where the conjugation is necessary for each batch of products. Another striking feature of the phage display is the ability to exploit the bacteriophages to display certain peptides that could help in crossing biological barriers efficiently [74]. Such phage display has been shown to assist the phages in overcoming physiological barriers such as the blood brain barrier. This is crucial for a therapeutic vector if the tumor of target is in the brain [75,76]. Another interesting application of phage display related to medicine is their proven role as vaccines at pre-clinical level [64]. Phage display is used to display viral or bacterial antigenic peptides on phage capsids followed by in vivo administration to induce an immune response [77,78]. Due to the inherent ability of phages to induce balanced innate and adaptive immunity, phage vaccines are shown to impart better synergistic immune response when compared to free subunit antigens, without the use of adjuvants [79]. Additionally, phage vaccines are considered as alternative to the well-known human virus-based vaccines due to their safety profile and non-infectious nature to mammalian cells. 

***Cargo capacity:*** Phages are proven to carry wide variety of cargoes that ranges from small dyes to large DNA molecules. Phages have efficiently carried diverse payloads such as fluorescent dyes, photosensitizers, QDs, other small nanoparticles, protein drugs, chemotherapeutic drugs, siRNA, CRISPR-Cas, and large mammalian gene expression cassette [80,81,82,83,84,85,86,87]. Due to their natural evolution in carrying their own large genomes, phage vectors can be manipulated to encapsidate large genes/genes of interest by synthetic genetic engineering techniques; further, due to safety concerns related to mammalian viral vectors, the safe-to-human phage vectors are now being considered as suitable alternatives. The wide range of cargo that phages could ferry projects the phages as a versatile carrier to treat various human diseases. 

## 7. Distinguishing Features of Phage Carriers for Cancer

Phage carriers as anti-cancer agents present great opportunities and offer greater beneficial outcomes which even surpass those of nanomaterial-based carriers. Phages can be chemically tailored to suit the purpose they are intended for, be it for anti-cancer therapeutics or as vaccines. In addition, phages can be genetically engineered to carry certain traits and do not require batch-by-batch chemical tailoring as in the case of nanoparticles. Due to the capsid’s nanosize and their resemblance to nanocarriers, the high surface-volume ratio helps to carry larger payloads as well as safeguarding the drug payload from biological degradation/inactivation, thus preserving the molecular functionality of the payload [88]. Drug cocktails can also be loaded into phage capsids that can help in treating drug resistant/heterogeneous tumors. In addition to acting as vector, the phages could guide the release kinetics of encapsulated/surface conjugated drugs. They can be engineered to modulate the release as per need and demand: slow and sustained, fast and pulsed, or external stimuli based. Ease of surface functionalization, either chemical or genetic (phage display), facilitates any number or type of moieties to be attached on the capsid surfaces that are available to external milieu [89]. Phage display can also help in a multivalent target approach where different peptides can be displayed on each capsid head, making them multivalent. This approach enhances selective targeting by manyfold and thus results in improved and precise therapeutic efficacy [90]. Another fascinating aspect is their ability to multifunction [91]. A single phage capsid can be loaded with varied functional agents such as other nanoparticles, photothermal agents, or photosensitizer, along with drugs to make them perform multiple applications in synergy. Macromolecular/molecular drugs are expelled by drug-resistant tumors easily by specialized evasion mechanisms whereas therapeutic phages due to their nanosize effect are not expected to be expelled so easily as they utilize endocytosis as mode of target cell entry. These exciting and beneficial features support the fact that phages can perform and fulfil the obligations vital for effectual therapeutic vectors with minimal pitfalls. 

## 8. Challenges Faced by Phages in Combatting Tumor

**Figure 2:**
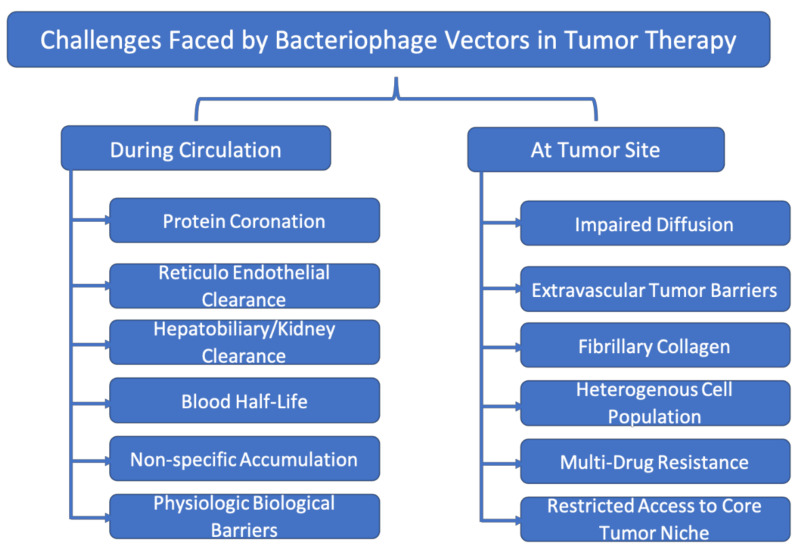
Challenges faced by cancer therapeutic bacteriophage vectors in biological systems.

Though we have discussed about the advantages of phage vector systems for cancer theragnostics, there are quite a few challenges that are yet to be addressed. The challenges faced by therapeutic phage vectors during circulation and at tumor sites are discussed below as well illustrated in Figure 2:

***Challenges faced during circulation:*** Once injected into the blood stream, the phages encounter numerous blood proteins that coronate on their surface. This coronation then signals to the RES system to clear the phages from circulation [92]. This phenomenon affects the blood half-life of phage vectors and thus their biodistribution and pharmacokinetics. With decreased half-life, the phage vectors lose their potential to reach and accumulate at the target site. The hepatobiliary and kidney clearance is the next major biological defense hurdle for the phages. Any foreign substances injected into blood stream encounter the hepatobiliary system that filters them and clears them from the system. This clearance also compromises the efficacy of therapy to great extent and may lead to non-specific drug-related toxicity [93]. The biochemical and biophysical barriers also impede the entry of phage vectors into organs such as the brain and pancreas [94]. Designing a smart system that can overcome these challenges can improve the overall efficacy of the phage carrier system. 

***Challenges after reaching the tumor:*** The major challenge faced by phages upon reaching the tumor site is related to diffusion. For instance, phages though they can reach the periphery of a tumor, cannot diffuse to the core due to high interstitial pressure. Though numerous neoangiogenic vessels are seen at the tumor site, due to their unconventional features as their dilated and collapsed nature, discontinuous blood supply and backflow, all account for the impaired diffusion of phage vectors. Apart from this, the core or certain regions of tumor are off-limits to the any therapeutic vector to access. As a result, cancer and stem cell populations at such sites can continually proliferate leading to systemic treatment failure [95]. To overcome such issues, combination therapy with vascular-modulating drugs can be carried out prior to phage vector exposure. The fibrotic stroma around most tumors also poses a hindrance to phage diffusion. To cross such barriers, fibrillary collagen matrix degrading enzymes can be co-administered with phage vectors. This enzyme can digest the fibrotic stroma, thereby paving the way for therapeutic vectors in reaching the tumor. Tumors, due to their complex biology, are highly heterogeneous; no two tumors of same organ/tissue origin remain similar genotypically/phenotypically. In addition, the tumor niche also has many non-tumor cellular counterparts, thereby leading to complexities. To tackle the tumor heterogeneity, combination therapy that addresses/modulates the tumor microenvironment and cellular components must be developed [96]. Multidrug resistance is also one of the hallmarks of many cancers that makes treatment difficult. These types of tumors have efficient efflux systems that rapidly clear therapeutics from their cytosol. Phage vectors that can tackle multidrug resistance by delivering the drugs to resistant variants and that overcome the other mentioned challenges simultaneously could provide enhanced support in the fight against cancer. 

## 9. Using Phages to Target and Regulate Tumor and Its Microenvironment

Many exciting phage therapeutic vectors are being developed and many are in pre-clinics now. The pre-clinical studies have proven that phages exhibit extended half-life, better pharmacokinetics, perfect specificity, on-demand drug release, and reduced off-site toxicity. For example, SP94-targeted virus like phage particles (VLPs) loaded with therapeutic drugs such as doxorubicin, cisplatin, and 5-fluorouracil is known to selectively kill Hep3B cancer cells in vitro. The same study also elaborated the use of the SP94-targeted VLPs in encapsulating a siRNA cocktail, that silenced the expression of the cyclin family members, inducing growth arrest and apoptosis of Hep3B cells. Notably, the same VLPs that co-displayed histidine-rich fusogenic peptide (H5WYG) and loaded with ricin toxin A-chain (RTA) promoted endosomal escape of the VLPs and killed target cancer cell populations without affecting the viability of control cells [97]. In another study, the M13 phage capsids were chemically modified for attachment of fluorophores and polyethylene glycol (PEG2k) without disturbing the binding ability of the phage-displayed antibody fragments to EGFR and HER2, the two important epidermal growth factor receptors seen overexpressed in breast cancer cells. The utility of these modified phages is demonstrated in the targeted imaging of breast cancer cells using multicolor fluorescence microscopy [98]. Further, chemically as well genetically engineered M13 bacteriophages were successfully utilized for intracellular delivery of exogenous proteins to human prostate cancer cells. The phages were made to display a biotin acceptor peptide (BAP) that functionally binds myriads of streptavidin-functionalized moieties and were successfully utilized for the delivery of two exogenous streptavidin bound proteins, GFP and HRP, into prostate cancer cells in vitro. The intracellular delivery of HRP was tested using HRP-dependent oxidation of indole-3-acetic acid (IAA) that is capable of producing peroxyl radicals that mediate cell killing [99]. In another interesting study, a refactored M13 bacteriophage that targets SPARC glycoproteins is utilized for targeted tumor imaging and therapy. The authors physically separated overlapping genes using a process known as refactoring. The redesigned genome was further genetically manipulated to display BAP and the phages were enzymatically biotinylated and conjugated to streptavidin-AlexaFluor dye to form M13-983-Alexa-phage, a targeted imaging probe carrying phage for tumor imaging applications. Additionally, the phages were packed with doxorubicin (DOX), a potent chemotherapeutic used extensively in the clinic. The release of the drug is facilitated bythe phage display of DKF motif, that is known to be recognized by cathepsin B, a lysosomal protease that is reported to be overexpressed in most prostate cancer cell lines [100]. Genetically engineered M13 phages that displayed two functional peptides, collagen mimetic peptide and streptavidin binding peptide, on their minor and major coat proteins, respectively, were prepared in another work. The resultant engineered phage functions as a therapeutic or imaging material to target degraded and denatured collagens in cancerous tissues. This work demonstrated that the engineered phages were able to efficaciously target and label abnormal collagens expressed on A549 human lung adenocarcinoma cells after conjugation with streptavidin-linked fluorescent agents [101]. In another interesting work, bacteriophage MS2 was developed as a targeted, multivalent photodynamic therapy vector for the treatment of Jurkat leukemia T cells. Each phage capsid was decorated with up to 180 porphyrins, a chemical agent capable of generating cytotoxic singlet oxygen upon illumination. The outer capsid surface was chemically modified to bind Jurkat-specific aptamers; the capsids were proven to target and selectively kill more than 76% of the Jurkat cells 20 min post PDT exposure [102]. Another exciting study utilized phage-nano assembly as drug delivery vehicles. In brief, nanosized poly(caprolactone-b-2-vinylpyridine) particles coated with folate-conjugated M13 bacteriophage encapsulating hydrophobic antitumor drug doxorubicin was prepared. The nano-phage structures were successfully utilized to target and treat KB cells that overexpressed folate receptors [103]. In one another work, a novel miR-122 delivery system based on MS2 phage that displays TAT peptide was developed. The miRNA has an anti-cancer effect whereas the surface display peptide enables cell penetration [104]. This delivery system has shown substantial advantages in terms of the vector’s biocompatibility and biodegradability, easy and quick preparation, and efficient anti-cancer effect.

**Figure 3:**
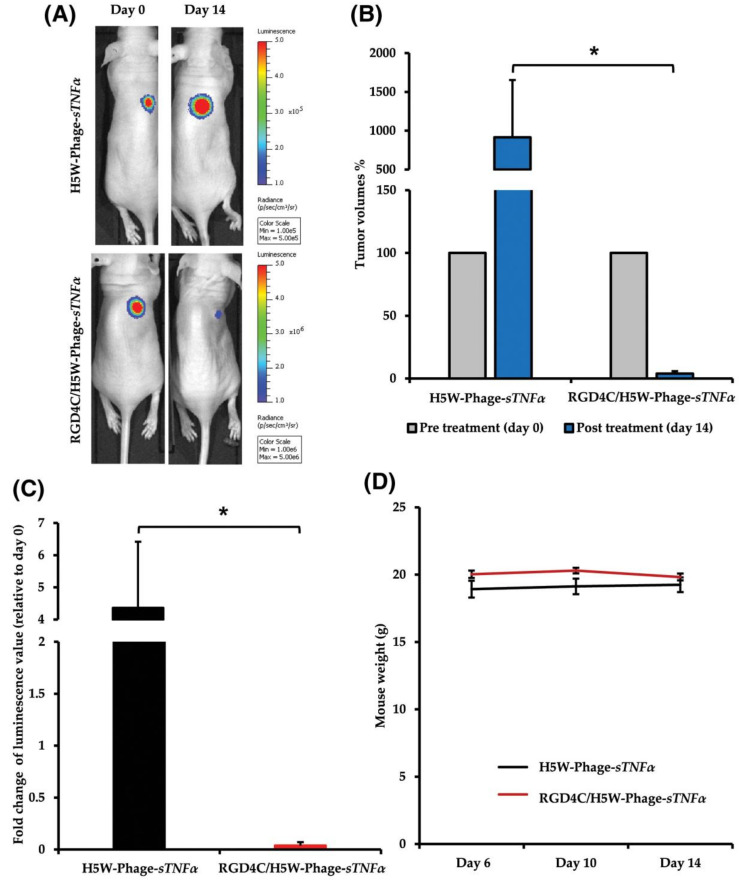
In vivo treatment of chondrosarcoma SW1353-bearing mice with intravenous administrations of RGD4C/H5W-Phage-sTNFα. Tumor-bearing mice were intravenously injected with RGD4C/H5W-Phage-sTNFα or non-targeted H5W-Phage-sTNFα vector. (**A**) Representative tumor-bearing mice imaged using the in vivo Bioluminescent Imaging System at day 0, before treatment initiation, and day 14, post-vector administration. (**B**) Average tumor volumes progression in each experimental group. (**C**) The luminescence values of tumors shown as fold change between pre-treatment day 0 and post-vector treatment day 14. (**D**) Average weights of SW1353 tumor-bearing mice. Adapted with permission from FASEB. * *p* < 0.05.

Gene delivery to mammalian cells has also been accomplished by phage vectors. In one such exciting research, the T4 phage was engineered to deliver genes; DNA molecules were translocated into emptied phage head and its outer surface was decorated with proteins fused to one of the outer capsid proteins. The T4 nanoparticles were efficiently targeted and delivered into antigen presenting dendritic cells, and offer a superior delivery system for recombinant DNA [105]. Another example for phage vector mediated gene delivery was attempted using a cancer cell bio-panning experiment. F5 phage antibody library was bio-panned along with SKBR3 cells to identify the phages that were able to bind the target. Next, to achieve gene delivery, F5 phages that exhibited successful target binding were then engineered to pack the GFP reporter gene cassette under the CMV promoter. These phages were efficiently endocytosed by the target cells; the GFP expression was seen only in the cells that overexpressed the ErbB2 receptor, indicating the feasibility of targeted gene delivery [106]. In another study, Tsafa et al. established a synergistic cancer chemo-virotherapy utilizing phage vector, AAVP—a cancer targeted, hybrid recombinant vector carrying AAV genome inside filamentous phage capsids. In addition, the vector carries two cargoes: a chemotherapeutic, DOX, and a suicidal gene cassette. The suicidal gene cassette codes for an HSVtk enzyme that is known to inhibit cell doubling and subsequently induce target cell death. The authors established an efficient synergistic cancer cell killing by the combined effect of Dox and the suicidal gene by utilizing the phage vector [107]. In yet another study by the same group, the investigators customized the phage capsid to display the RGD4C ligand on the pIII minor coat proteins for targeting purposes and a human tumor necrosis factor alpha (TNFα) therapeutic transgene was fused into the phage genome. When chondrosarcoma SW1353 cells were exposed to the phage vector encoding a TNFα transgene, distinct targeted cell killing concomitant with the high expression of TNFα and apoptosis-related genes was witnessed. In vivo, chondrosarcoma implant-carrying mice exhibited inhibition of tumor growth post phage vector administration (Figure 3) [108]. In another study, phage mediated gene replacement therapy was attempted. For this purpose, cancer cell targeted phage vectors were genetically reconstructed to carry 10 kb of CRISPR-Cas9 sequence. When these phage vectors were co-incubated with the target lung adenocarcinoma cells, the phage vectors successfully mediated the transgene Cas9 expression. In addition, the authors have also proven the targeted silencing of p53 gene expression when a p53 gRNA is included in the phage gene construct [109]. This was a proof-of-concept study that proved that phage vectors can be utilized for gene silencing by shipping CRISPR-Cas to target mammalian cells intracellularly.

**Figure 4:**
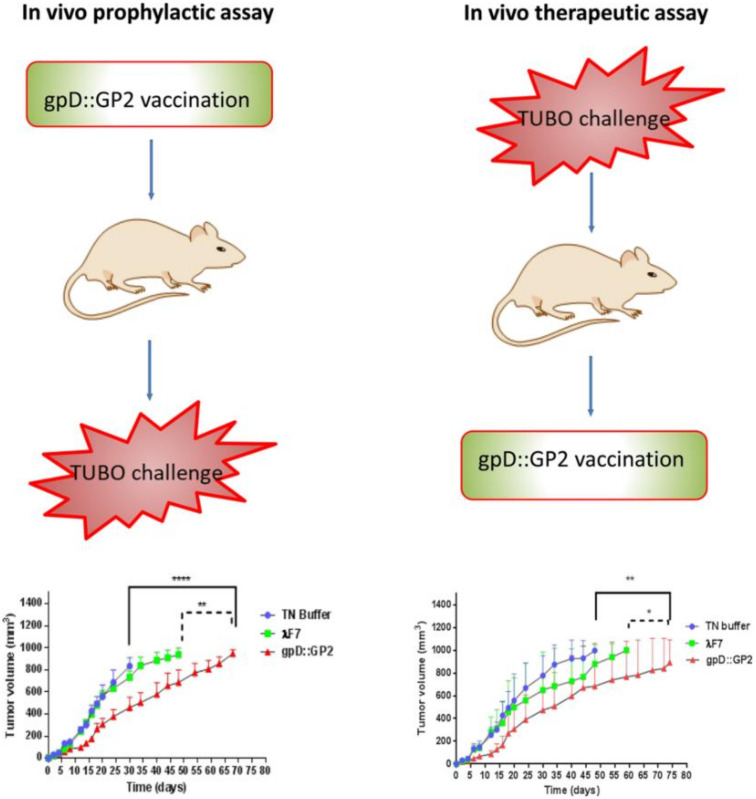
A schematic summary of the breast cancer vaccine study (gpD::GP2) in a TUBO tumor model of BALB/c mice. Adapted with permission from NPG. * *p* < 0.05, ** *p* < 0.01 and **** p < 0.0001; denotes significant difference from the control groups.

With respect to phage mediated tumor vaccine development, a T4 phage particle that displays mFlt4 on the surface was constructed and evaluated as a recombinant vaccine. The T4-mFlt4 recombinant vaccine exhibited antitumor activity stimulating autoantibodies against the antigen. Vaccine-treated LLC-derived tumors carrying mice exhibited prolonged survival when compared to control animals. In addition, the vaccine also limited the lymphangiogenesis and tumor metastasis in treated mouse models [110]. In another study, M13K07 anticancer vaccine phage capsids were genetically modified to display immunogenic cancer epitope Δ16HER2. The phage capsids exhibited good immune tolerance along with efficient anti-Δ16HER2 humoral immune response upon immunization in vivo in the Δ16HER2 mice model [111]. In another example, Iwagami et al. evaluated the preventive effects of ASPH antigen carrying lambda phage vaccine constructs against ASPH expressing murine liver tumor model. Post immunization, mice pre-treated with phage vaccines exhibited anti-tumor activity upon subcutaneous tumor implantation. A good amount of antigen specific humoral response was generated. The study also documented the infiltration of lymphocytes into tumors, indicating successful prophylactic and therapeutic vaccination [112]. In another study, the authors developed an immunogenic bacteriophage-based vaccine to provoke cytotoxic T lymphocyte activity in an HER2/neu expressing mice tumor model. The outcome of the research implied that phage displaying GP2 as a fused peptide to the gpD phage capsid protein stimulated a robust CTL response. Besides, the chimeric phage protected the mice against HER2/neu-positive tumor challenge in both prophylactic and therapeutic settings (Figure 4). These phage-based vaccine developments provide a rationale for the use of phages as antigen carriers as well as for safe and efficacious cancer preventive measures [112]. 

With the examples noted above, it is possible to state that phage vectors are perfect options to treat and diagnose cancer, with better outcomes. More such examples are tabulated in Table 1. These results cumulatively indicate that phage-based therapeutic vectors are promising, selective, and efficient tools for targeted cancer therapy. 

## 10. Future Perspective

Bacteriophages, being one of the most important constituents of the human body with a vital role in bacterial population dynamics and host immune response modulation, are generally considered safe. Bacteriophage-based anti-cancer approaches can be made robust with the assistance of phage display technology. The phages can be made to display peptides that could help in selective target cell binding or improving blood circulation time or modulating the extracellular matrix or in regulating/interfering with cancer progression mechanisms. Through these direct or indirect tumor modulating features, phages can intensify the effects of anti-cancer strategies. Another criterion that needs better understanding is how to reduce the immune response mediated phage clearance from the system. This is a real roadblock when considering phage as a theragnostic vector. More studies are necessary to overcome the potential host immune response against the phage vectors. In addition, it is also essential to acquire a deep understanding of the methods to optimize the use of bacteriophages for cancer theragnostic applications. 

Designing and tuning phage vectors biochemically or genetically as per need for a personalized therapy is not only enticing but feasible. Basic understanding of tumor niches and exploiting them for design of personalized theragnostic vectors would allow such preparations to function extremely well in the tumor environment: for example, modifying phages to release drugs in acidic environments, modifying phages to fluoresce in a hypoxic niche, and utilizing suitable targeting with phage display techniques to make phages smart vectors/probes for cancer theragnostic applications. With multiple diversecargo-carrying ability in one vector, phages can also function as multifunctional vectors, performing multi-tasking at any given time. In additional, phages that target tumor niches such as degraded ECM or tumor endothelia and/or cancer fibroblasts are exciting to research as regards their potential utilization for combinational therapy, with phage-chemotherapy leading to more focused and synergistic treatment regimes. As this field is still in its infancy and yet to be explored to its threshold, one expects a remarkable increase in scientific innovation. It is therefore essential to perform active research, more proof-of-principle, pre-clinic, and clinical trials and to generate data to support the beneficial therapeutic effect of chaperoning phage vectors in terms of extravasating from tumor blood vessels, migrating inside the tumor parenchyma, responding to tumor cues, and then reaching the tumor cytosol. This in principle would be an added value of delivery in oncolytic phage-based therapy for clinical use in future years.

## Figures and Tables

**Table 1:** List of phages utilized for cancer theragnostic.

Phage	Ligand Insertion Method	Ligands	Cargo	Application
MS2 Virus like particle [97,102,113,114]	Phage display	SP94 peptide (for targeting) H5WYG (for endosomal escape)	QDs, siRNA, DOX, Ricin	Targeted cargo delivery into Hep3B cancer cells
	Chemical method	TAT peptide (for cell penetration)	Antisense RNA	Antisense RNA delivery system
	Chemical method	Transferrin (for targeting)	Antisense RNA	Targeted killing of leukemia cells
	Chemical method	Aptamer (for targeting)	Porphyrins	Targeted delivery of photodynamic agents to cancer cells
M13 [97,98,99,100,101,103,115,116,117,118,119]	Phage display	single-chain antibody fragments (scFvs) (for targeting)	Fluorophore	Cancer marker imaging agents
	Phage display	Ypep (for targeting) Biotin Acceptor peptide (for cargo loading)	Streptavidin-GFP, Streptavidin-HRP	Biotinylated phages for intracellular delivery of exogenous proteins
	Phage display	SPARC binding peptide (targeting) Biotin Acceptor peptide (for cargo loading) Peptide motif DFK (for facilitating DOX release)	Streptavidin- Alexa Fluor, DOX	Tumor cell imaging & drug delivery
	Phage display	Collagen mimetic peptide (CMP) (for targeting collagen) Streptavidin binding peptide motif (for cargo loading)	Streptavidin-Alexa Fluor®488	Collagen targeted cancer imaging
	Chemical method	Folic acid (for targeting)	-	Cancer cell imaging
	Chemical method	Folic acid (for targeting)	DOX	Drug delivery vehicle
	Chemical method	-	FITC, RBITC	Phage based intracellular pH indicator
	Chemical method	FGF2 (for targeting)	GFP/3-Gal gene	Intracellular gene delivery
	Phage display	RGD (for targeting)	-	Intracellular gene delivery
T4 [105,110]	Phage display	mFlt4 protein on T4 surface using capsid surface Soc and Hoc bipartite expression and display	-	Cancer immunotherapy
	Phage display	CPPs, (DEC)205, CD40	Reporter genes, vaccine candidates, functional enzymes	In vitro and in vivo delivery of genes and proteins
Lambda [120]	Chemical method	Holotransferrin (for targeting)	GFP gene	Targeted bacteriophage-derived gene nanocarriers into eukaryotic cells

## Data Availability

Not applicable.
